# Chain Extension and Synergistic Flame-Retardant Effect of Aromatic Schiff Base Diepoxide on Polyamide 6/Aluminum Diethylphosphinate Composites

**DOI:** 10.3390/ma12142217

**Published:** 2019-07-10

**Authors:** Tianxiang Liang, Jianan Cai, Shumei Liu, Hualin Lai, Jianqing Zhao

**Affiliations:** 1 School of Materials Science and Engineering, South China University of Technology, Guangzhou 510640, China; 2 Shenzhen Halcyon New Materials Co., Ltd., Shenzhen 518116, China

**Keywords:** polyamide 6, chain extension, flame retardancy, Schiff base, mechanical properties

## Abstract

A way to suppress the deterioration in mechanical properties of polyamide 6 (PA6) is required, especially with high loading of flame retardants in the matrix. In this study, a novel aromatic Schiff base diepoxide (DES) was synthesized. It exhibited an efficient chain extension effect on PA6 and a synergistic flame-retardant effect with aluminum diethylphosphinate (AlPi) for PA6. The PA6 composite with 16 wt.% AlPi only passed UL-94 V-0 rating at 1.6 mm thickness, while the combination of 1.5 wt.% DES with 13 wt.% AlPi induced PA6 to achieve a UL-94 V-0 rating at 0.8 mm thickness. The tensile, flexural, and Izod notched impact strengths were increased by 16.2%, 16.5%, and 24.9%, respectively, compared with those of V-0 flame-retarded PA6 composites with 16 wt.% AlPi. The flame-retarded mechanism of PA6/AlPi/DES was investigated by cone calorimetry and infrared characterization of the char residues and pyrolysis products. These results showed that DES had a synergistic effect with AlPi in condensed-phase flame retardation by promoting the production of aluminum phosphorus oxides and polyphosphates in the char residues.

## 1. Introduction

Polyamide 6 (PA6) is widely used as an engineering plastic due to its excellent mechanical and thermal properties. However, the flammability and molten drop phenomenon of PA6 during combustion limits its application in many places. The incorporation of flame retardants is an efficient way to address these drawbacks [1,2]. Halogenated flame retardants are effective for polyamide, but they release corrosive gases and poisonous smoke during combustion. Various halogen-free flame retardants, such as magnesium hydroxide [3,4,5], red phosphorus [6,7], melamine cyanurate [8,9,10], and melamine polyphosphates [11,12,13], have been developed and commercialized [1]. Many efforts have also been devoted to novel flame retardants, such as halloysite nanotubes [14,15,16], organoclay nanocomposite [17], and carbon nanotubes [18,19]. A novel flame retardant, aluminum diethylphosphinate (AlPi), has been proven to be an effective flame retardant for PA6 [20,21,22]. The addition of 20 wt.% AlPi in PA6- or glass-fiber-reinforced PA6 achieved the UL-94 V-0 rating at 3.2 mm thickness [23,24]. However, to achieve the desired flame retardation level, the high loading of flame retardants is usually required, which deteriorates the mechanical performance of PA6 [23,25,26]. In addition, the presence of impurities and moisture, especially flame retardants, in PA6 matrix induces the decomposition of the molecular chains during high-temperature processing, leading to a decline in mechanical properties of the materials. To solve the problems above, chain extenders have been developed and have proven to be an effective additive for suppressing this mechanical deterioration [27,28]. Epoxide and oxazoline type chain extenders have been revealed to have high reactivity with amino and carboxyl end-groups of polyamide, which increases the molecular weight and mechanical properties of polymers [29,30,31,32].

Innovative ideas have been proposed to combine flame retardancy and chain extension. Flame-retardant structures, such as isocyanurate [21], cyclotriphosphazene [26], and 9,10-dihydro-9-oxa-10-phosphaphenanthrene-10-oxide (DOPO) [25,33], have been introduced into the chain extenders, which enhanced the flame retardancy and mechanical properties of PA6 composites simultaneously [34,35,36]. The molecular design of the chain extenders strategy mentioned above effectively combines flame retardancy and chain extension to endow the composites with good overall properties.

Recently, some novel high-temperature, cross-linkable copolymers with flame retardancy and anti-dripping performances have been reported [37,38,39,40,41]. Cross-linkable groups, such as phenylethynyl groups [37,38,39], phenylmaleimide groups [40], azobenzene groups [41], and aromatic Schiff base groups [42], were introduced into the polyester molecular chains in the backbone or as pendant groups by copolymerization. These copolymers were stable during melt processing, but cross-linked rapidly at a proper temperature between the melting and decomposition temperatures, which endowed the copolymers with flame retardancy and anti-dripping properties. However, it was necessary to copolymerize the cross-linkable groups into the molecular chains, which has a high cost and is not applicable to the production of modified engineering plastics. Low-cost additives which can bring about this cross-linkable property of polymers are desired.

**Scheme 1:**
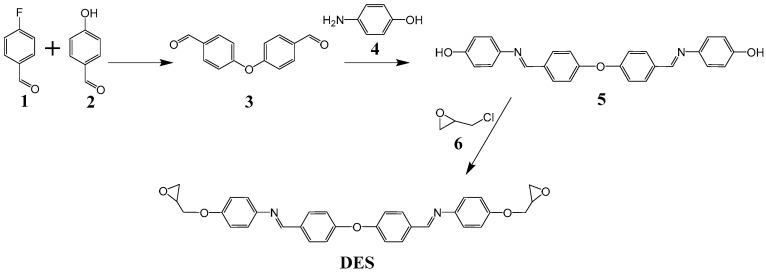
The synthetic route of the diepoxide (DES).

To achieve this objective, one feasible and economical way is to incorporate cross-linkable groups into the PA6 backbone by chain extension reactions during the melt processing of PA6. The cross-linkable groups, like Schiff bases, are first introduced into the chain extenders. Compared with the flame-retardant units reported previously [21,25,26,33], Schiff bases have the features of simple molecular structure, phosphorus-free, thermal stability, and easy synthesis, and have been revealed to be a potential self-crosslinking unit for achieving flame-retardant and anti-dripping performances [43,44,45,46]. Chain extenders containing Schiff bases are expected to enhance the flame retardancy and mechanical properties of PA6 composites simultaneously. Such additives are economical and practical to the production of flame-retarded PA6 at a large scale. In this manuscript, a novel diepoxide compound (DES, Scheme 1) containing aromatic Schiff base groups was synthesized and used as a chain extender and flame-retarded synergist in flame-retarded PA6/AlPi composites. The chain extension effect, mechanical performance, flame retardancy, forced combustibility, and thermal degradation of the obtained flame-retarded PA6 composites were investigated. The char residues and pyrolysis gases were analyzed in-depth to explore the flame-retarded mechanism.

## 2. Materials and Methods 

### 2.1. Materials

**Scheme 2:**
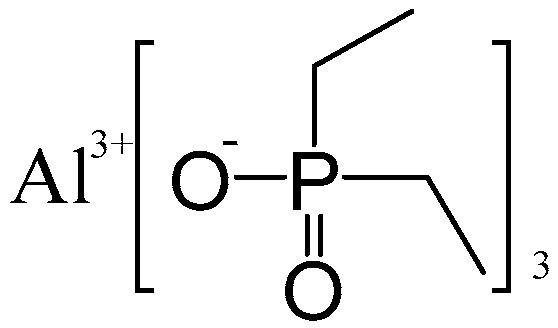
The chemical structure of AlPi.

PA6 (YH 800) was supplied by Yueyang Baling Shihua Chemical & Synthetic Fiber Co. Ltd. (Yueyang, China). AlPi (Exolit OP 1230) was supplied by Clariant Chemicals Co. (Frankfurt, Germany). The chemical structure of AlPi is shown in Scheme 2. 4-Fluorobenzaldehyde, 4-hydroxybenzaldehyde, 4-aminophenol, epichlorohydrin, and potassium carbonate were purchased from Shanghai Aladdin Bio-Chem Technology Co. (Shanghai, China). Chemicals mentioned above were used as received. 

### 2.2. Synthesis of DES

4-Fluorobenzaldehyde (named as 1, 0.1 mol), 4-hydroxybenzaldehyde (2, 0.1 mol), potassium carbonate (0.1 mol), and N, N-dimethylacetamide (100 mL) were added into a three-necked flask, and the mixture was stirred for 5 h at 110 °C under a nitrogen atmosphere. Insoluble matter was removed by filtration and the filtrate was evaporated under reduced pressure. The product was recrystallized from ethyl acetate/ethanol to give bis(4-formylphenyl) ether (3) as a white powder with a yield of 95%. 

Bis(4-formylphenyl) ether (3, 0.1 mol), 4-aminophenol (4, 0.2 mol), and anhydrous ethanol (400 mL) were added into a three-necked flask, and the mixture was stirred for 5 h at 50 °C under a nitrogen atmosphere. The mixture was cooled to room temperature, and the resulting precipitate was filtered and washed with water and ethanol three times each to give intermediate compound 5 as a white solid with a yield of 90%.

Compound 5 (0.034 mol), potassium hydroxide (0.034 mol), and anhydrous ethanol (400 mL) were added into a three-necked flask. The mixture was stirred until completely dissolved before epichlorohydrin (6, 0.5 mol) was added. The mixture was stirred for 3 h at 60 °C under a nitrogen atmosphere. Next, potassium hydroxide solution (40 wt.%) was dropwise added to the system. The reaction proceeded at 75 °C for 5 h. The mixture was cooled to room temperature, and the resulting precipitate was filtered and washed with water and ethanol three times each to give DES as a white solid with a yield of 88%.

The synthesized products were characterized by ^1^H nuclear magnetic resonance (NMR) spectra (Figures S1–S3) and Fourier transform infrared (FTIR) spectra (DES, Figure S4).

### 2.3. Instruments and Methods

^1^H-NMR spectra were measured on an Avance Digital 600MHz spectrometer (Bruker, Karlsruhe, Germany) with chloroform-*d* or DMSO-*d_6_* as solvent. FTIR spectra were measured on a Vertex70 FTIR spectroscopy (Bruker, Karlsruhe, Germany). Thermogravimetric analysis (TGA) was carried out on a TG-209F1 thermogravimetric analyzer (Netzsch, Selb, Germany) under a nitrogen atmosphere at a heating rate of 10 °C min^−1^ from 30 °C to 700 °C. Thermogravimetric Fourier transform infrared spectroscopy (TGA-FTIR) was done using a Netzsch STA 449C TG instrument connected to a Bruker Tensor 27 FTIR. The samples were heated from 30 °C to 700 °C at a heating rate of 20 °C min^−1^. Differential scanning calorimetry analysis (DSC) was performed using a DSC-204F1 (Netzsch, Selb, Germany) at a heating rate of 10 °C min^−1^ from 30 °C to 700 °C. Before the DSC measurement, the samples were heated to 260 °C rapidly and maintained at 250 °C for 5 min to erase their thermal history, and then cooled to 30 °C at a rate of 10 °C min^−1^. 

Limiting oxygen index (LOI) tests were carried out on an FTA-SC48 oxygen index instrument (Fire Testing Technology, East Grinstead, UK) according to the ISO 4589-2 (sample size: 150 × 10 × 4 mm^3^). Vertical burning tests were determined using a UL94-SC50 flame chamber (Fire Testing Technology, East Grinstead, UK) according to UL94-2009 (sample size: 127 × 12.7 × 1.6 or 0.8 mm^3^). The forced-flaming performance was characterized by the cone calorimeter (Fire Test Technology, East Grinstead, UK) referring to ISO5660-1-2000 under an external heat flux of 35 W/m^2^ (sample size: 100 × 100 × 5 mm^3^). The morphology of residues was measured on a TM3000 scanning electron microscope (SEM) (Hitachi, Tokyo, Japan) equipped with an energy dispersive X-ray spectroscope SwiftED3000 EDX (Hitachi, Tokyo, Japan).

Tensile and flexural strengths of specimens were determined using an AGS-10KNI universal electronic tensile testing machine (Shimadzu, Kyoto, Japan) according to ISO 527-2012 and ISO 178-2010. Izod notched impact strength was determined using a Zwick5113 pendulum machine (Zwick Roell, Ulm, Germany) according to ISO 180-2001. Melt flow rate (MFR) was measured at 250 °C and 2.16 kg load using an XNR-400B melt flow tester (Shipeng Detection Equipment Co., Ltd. Chengde, China) according to ISO 1133-1-2011.

### 2.4. Processing and Specimen Preparation

Chain extension effect was measured on a Haake Rheomixer 600 (Thermo Electron Corporation, Karlsruhe, Germany). 50 g of well-dried PA6 (100 °C for 6 h) was added into the mixing chamber of the Haake Rheomixer for 30 s. The rotor speed was set at 50 rpm and the temperature was 230 °C. The predesigned amount of DES was then added to the PA6 melt, as presented in Table 1. The melt torque during mixing was recorded to measure the extent of the reaction. Amino and carboxyl end-group contents of PA6 samples were evaluated by the Waltz–Taylor titration method [47].

The PA6/AlPi composites were prepared with the incorporation of 13 wt.% or 16% AlPi in PA6 matrix. The PA6/AlPi/DES composites were prepared with the incorporation of 13 wt.% AlPi and the predesigned amountof DES (see Table 1). The mixture of PA6, AlPi, and DES was melt-kneaded and extruded into pellets in a twin-screw extruder (L/D = 40, Labtech Engineering, Samutprakarn, Thailand) with a cylinder temperature of 230 to 240 °C at a screw speed of 40 rpm. The resultant pellets were dried at 100 °C for 6 h and then injection-molded into specimens for measurement of flame-retardancy and mechanical properties (EC75-NII, Toshiba, Tokyo, Japan) at an injection temperature of 250 °C.

## 3. Results and Discussion

The target compound DES was synthesized according to the reported procedures with some modification [25,48], and characterized by ^1^H NMR and FTIR spectroscopy. The synthetic routes of DES are summarized in Scheme 1. The melting point of DES was found to be 237 °C by DSC measurement (Figure S5). The initial decomposition temperature at 5% weight loss (*T_5%_*) of DES is evidenced at 312 °C from the TGA curve (Figure S5), with 54 wt.% char residues at 700 °C. The decomposition temperature was higher than the processing temperature of PA6, meaning that DES has good thermal stability during melt processing.

### 3.1. Chain Extension Effect

**Figure 1:**
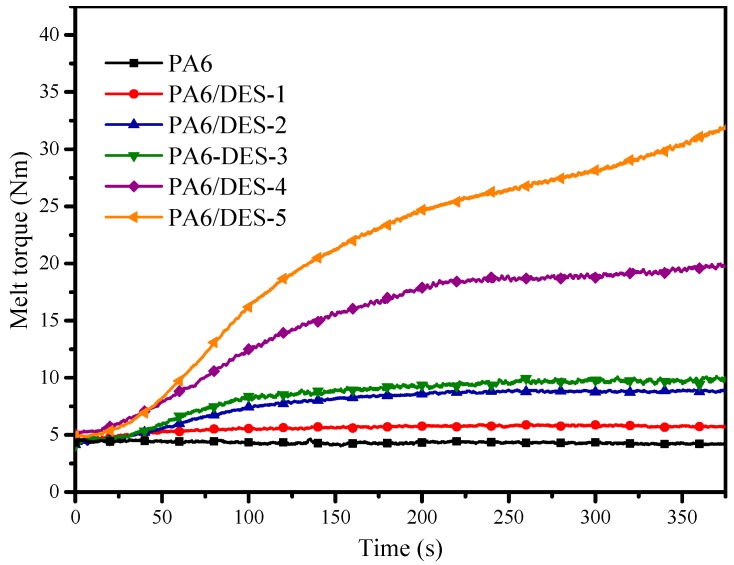
Melt torque of chain-extended PA6 with different DES contents.

The epoxide rings in the structure of DES can react with the amino and carboxyl end-groups of PA6 to elongate the macromolecular chains, leading to enhanced melt viscosity [33,36,49,50]. Chain extension effect is evaluated by measuring the melt torque on a Haake Rheomixer. As seen in Figure 1, the melt torque of PA6 increased incrementally with the increase of DES content. The addition of 2 wt.% DES improved the equilibrium melt torque by more than 50% compared with neat PA6. The torque values remained constant with the prolonging of reaction time, indicating that no thermal degradation occurred. However, it should be noted that over 4 wt.%, DES boosted the equilibrium melt torque significantly, due to the generation of branching or cross-linking structures. Chain extending reactions produced secondary hydroxyl groups, and these hydroxyl groups further reacted with the excessive epoxy groups, leading to branching or even cross-linking [51].

**Figure 2:**
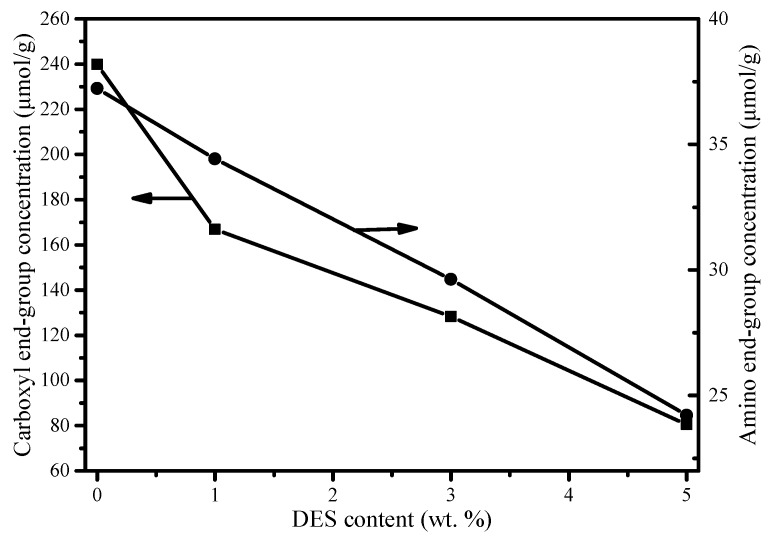
Carboxyl and amino end-group concentrations of chain-extended PA6 with different DES contents.

During the coupling reaction in the molten state, chain extenders consumed the amino and carboxyl end-groups of PA6 molecular chains, so the concentration of the end-groups was detected to decline. As presented in Figure 2 and Table S1, the concentrations of amino and carboxyl end-groups in chain-extended PA6 decreased concurrently. It is noteworthy that there exist much more carboxyl end-groups in PA6. The concentration of carboxyl end-groups declined faster than that of amino end-groups, because of their higher reaction probability.

### 3.2. Mechanical Properties and Crystallinity

The mechanical properties of neat PA6 and its composites with different loading levels of AlPi and DES were investigated. As shown in Table 2, the tensile, flexural, and Izod notched impact strengths of PA6/AlPi composites deteriorated simultaneously compared with neat PA6, due to the poor compatibility between AlPi and the polymer matrix [23,25,26]. Additionally, the melt flow rates also decreased with the incorporation of AlPi. However, the incorporation of a small amount of DES suppressed the mechanical deterioration, and the strengths were enhanced gradually with the loading of DES. For the PA6/AlPi composite system with 13 wt.% AlPi, only 0.5 wt.% DES enhanced the mechanical properties of PA6/AlPi composites, which approached the properties of neat PA6. Furthermore, PA6/AlPi/DES-1.5 achieved a tensile, flexural and Izod notched impact strength of 63.9 MPa, 97.6 MPa, and 7.2 kJ/m^2^, presenting enhancements of 13.4, 13.3, and 12.7% compared with those of PA6/AlPi-13 composites, respectively. The improvement in mechanical strengths is primarily attributed to the chain extension effect of DES in the PA6 matrix. The coupling reaction between the epoxide groups of DES and amino or carboxyl end-groups of PA6 improved the macromolecular weight of PA6, which increased the entanglement and friction of chain segments, and the mechanical properties [36].

Additionally, the significant descent of the melt flow rates provides further strong evidence for the chain extending reactions between PA6 and DES. As shown in Table 2, the melt flow rates declined from 11.8 g/(10 min) of PA6/AlPi-13 to 2.3 g/(10 min) of PA6/AlPi/DES-1.5, indicating that the melt viscosity and molecular weight of the composites were remarkably enhanced. It is noteworthy that the melt fluidity of all samples was sufficient in practice for injection molding. The tensile and flexural strengths of PA6/AlPi/DES-2 declined slightly compared with those of PA6/AlPi/DES-1.5, implying that the concentration of 1.5 wt.% DES in PA6 reached saturation for the coupling reaction.

**Figure 3:**
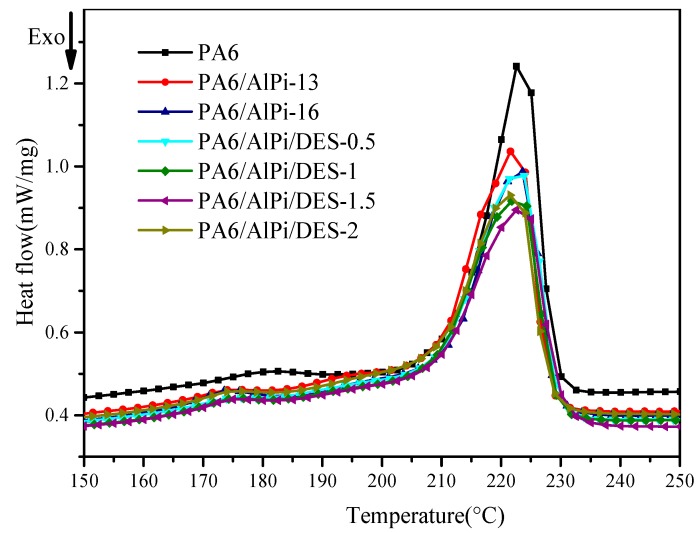
The DSC thermograms of neat PA6 and flame-retarded PA6.

To investigate the influence of DES and AlPi on the crystallinity of PA6 matrix, DSC analysis was conducted, and the thermograms of neat PA6 and PA6 composites are presented in Figure 3. The thermograms show that the addition of AlPi and DES did not obviously change the melting temperature (*T_m_*), but decreased the melting enthalpy (Δ*H_m_*) gradually. This means that the crystalline form of PA6 remained unchanged, and the crystallinity of specimens was decreased upon the addition of AlPi and DES.

The degree of crystallinity (*X_c_*) is determined by the following Equation (1):

(1) $$X_{c}=\frac{\Delta H_{m}}{\Delta H_{m}^{0}}\times \frac{1}{W_{f}}\times 100\%,$$

where Δ*H_m_* is the melting enthalpy of PA6 composites, *W_f_* is the content of crystalline material (PA6) in the composites, and $\Delta H_{m}^{0}$ is the melting enthalpy of fully crystalline PA6, the value of which is 190 J/g [52].

The *T_m_*, Δ*H_m_*, *W_f_*, and *X_c_* are summarized in Table 3. The crystallinity of PA6 composites descended with the loading of DES, implying that the chain extension hinders the crystallization of PA6 molecular chains [53]. The drop of crystallinity produces more disordered amorphous phase for absorbing deformation energy, leading to larger impact strength.

### 3.3. Flame Retardancy

Neat PA6 is a flammable material with burning drips during combustion, so it does not pass the UL-94 test. AlPi is an effective flame retardant for PA6, and the LOI values increase from 24.2% of neat PA6 to 29.3% of PA6/AlPi-13 and 31.8% of PA6/AlPi-16, respectively. However, the specimen of PA6/AlPi-13 produces burning drips in the vertical burning test, and achieves only a UL-94 V-2 rating at 1.6 mm thickness. The specimen achieves a UL-94 V-0 rating at 1.6 mm thickness unless the content of AlPi increases to 16 wt.%. 

In this work, the PA6/DES composites were flammable even with 5 wt.% DES. However, DES was proven to be conducive to the flame retardancy of PA6/AlPi composites. The LOI and UL-94 tests were performed to investigate the flame retardancy of the PA6/AlPi/DES composites. The results are listed in Table 4. With the addition of 13 wt.% AlPi, the LOI values increased significantly from 29.3% to 35.4% of PA6/AlPi/DES-2. Additionally, the test specimen of PA6/AlPi/DES-0.5 showed no burning drips, self-extinguished rapidly, and achieved a UL-94 V-0 rating at 1.6 mm thickness with only 0.5 wt.% DES. This means that the proportion of AlPi was remarkably reduced with small amounts of DES in flame-retarded PA6 composites, which is beneficial to mechanical properties. Moreover, the specimen of PA6/AlPi/DES-1.5 containing 1.5 wt.% DES passed the V-0 rating of the UL-94 test at 0.8 mm thickness. Such results indicate that the synergistic effect of AlPi and DES affords efficient flame-retardant properties. It is noteworthy that the specimen of PA6/DES-2 composites containing 2 wt.% DES was flammable and did not pass the UL-94 test. Consequentially, the tensile, flexural, and Izod notched impact strength of PA6/AlPi/DES-1.5 increased by 16.2%, 16.5%, and 24.9% compared with those of the V-0 flame-retarded specimen PA6/AlPi-16, respectively. Despite the absence of any traditional flame-retardant structures (like DOPO units), DES improved the flame retardancy of PA6, which strongly proves that the self-crosslinked ability of aromatic Schiff base groups at high temperature brings about a significant improvement in flame retardation [42].

### 3.4. Cone Calorimeter Test

**Figure 4:**
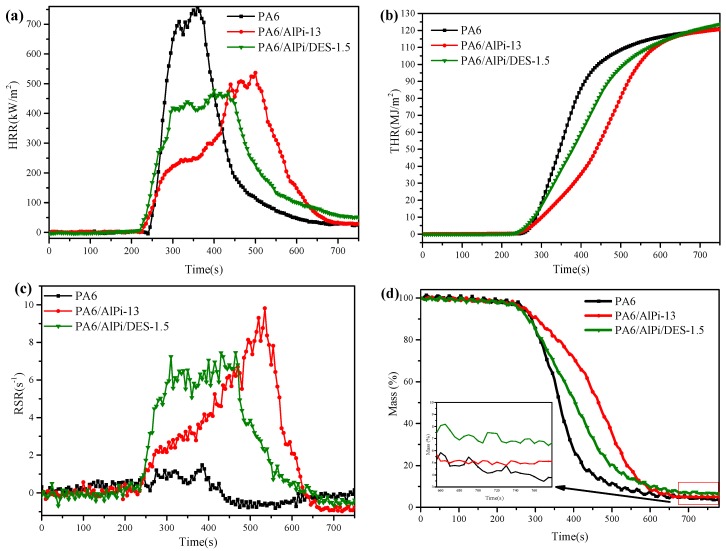
(**a**) Heat release rate, (**b**) total heat release, (**c**) rate of smoke release, and (**d**) mass loss curves of neat PA6 and flame-retarded PA6 in cone calorimeter test.

To further investigate the combustion behavior and flame-retarded mechanism, cone calorimetry measurement was carried out and the results are shown in Figure 4. The important parameters of cone calorimeter measurement are summarized in Table 5. Figure 4a,b presents the curves of heat release rate (HRR) and total heat release (THR) of neat PA6, PA6/AlPi-13, and PA6/AlPi/DES-1.5, respectively. Compared with the other curves, the HRR curve of PA6/AlPi/DES-1.5 presents a broad steady platform. The THR values of all samples were close, but the heat release of flame-retarded PA6 was much slower, which reduced the risk of conflagration. The peak HRR value of flame-retarded PA6 is much lower than that of neat PA6 as well. Compared with neat PA6, the peak HRR value of PA6/AlPi-13 was decreased by 29%. Moreover, PA6/AlPi/DES-1.5 shows a peak HRR of 478.0 kW/m^2^, with a 37% decrease compared to that of neat PA6. Time to ignition (TTI) of specimens is observed in Figure 4a. Fire ignition of the composites containing AlPi occurred about 20 s earlier than that of neat PA6 because the decomposition of AlPi stimulates the catalytic-degradation of PA6 matrix [25,26,54].

The curves of smoke release rate (RSR) of the specimens are presented in Figure 4c. In correlation with the total smoke release (TSR) values in Table 5, flame-retarded PA6 composites exhibited large RSR values, which is consistent with the gaseous-phase mechanism of AlPi. Phosphorus-containing volatiles decomposed from AlPi diffused into gaseous phases to quench the free radicals, leading to incomplete burning and plentiful smoke release [24,55,56]. Correspondingly, neat PA6 exhibited lowest smoke release, because a large number of combustible gases are released during the combustion process and fully burn in the cone calorimetric test. Additionally, small amounts of DES in PA6 matrix decreased the RSR and TSR slightly, and increased residual char, indicating that DES mainly acts in the condensed-phase flame retardation without a negative effect on the gaseous-phase flame retardation of AlPi. 

The mass loss curves in the cone calorimeter test are presented in Figure 4d, and the mass of residual char is summarized in Table 5. Compared with neat PA6, the mass of residual char of PA6/AlPi-13 and PA6/AlPi/DES-1.5 were increased by 42% and 86%, respectively. It is known that the inclusion of AlPi promotes the formation of char layer [57,58]. A small amount of DES in PA6 matrix increased the mass of residual char significantly, suggesting that the aromatic Schiff base groups of DES have a synergistic effect in the char formation of PA6 composites.

### 3.5. Residue Analysis 

**Figure 5:**
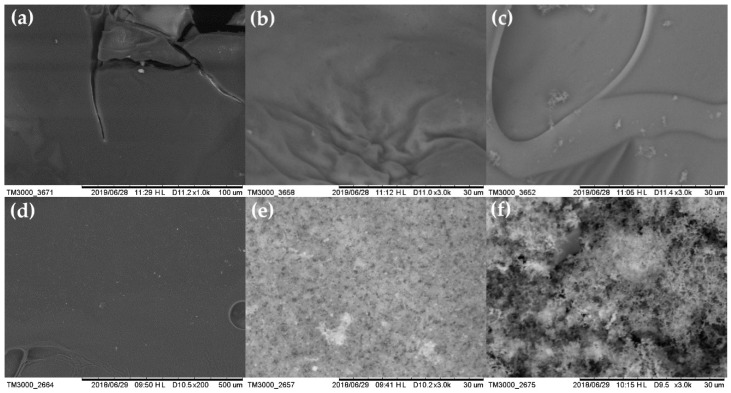
SEM images of residues for neat PA6 ((**a**): outer surface, (**d**): inner surface), PA6/AlPi-13 ((**b**): outer surface, (**e**): inner surface), and PA6/AlPi/DES-1.5 ((**c**): outer surface, (**f**): inner surface).

To get more detailed insight into the char residues, the micromorphology of the outer and inner char residues was investigated by SEM. As shown in Figure 5a–c, all residual samples exhibited a compact and smooth outer surface. For the residue of neat PA6, the inner surface was compact as well, as shown in Figure 5d. However, Figure 5e shows that the residue of PA6/AlPi-13 had a loose and porous inner surface, indicating that PA6/AlPi composite is a char-formed flame retardant system with few gases release during combustion [55]. Furthermore, as presented in Figure 5f, the residual char of PA6/AlPi/DES-1.5 shows looser and more porous inner surface. Additionally, the residual mass of PA6/AlPi/DES-1.5 increased by 31.4% compared with PA6/AlPi-13, and the residual char appeared more intumescent at macroscale. This intumescent char with a compact and smooth outer surface is a good barrier for heat and mass transfer, and thus provides a better flame shield to improve the flame retardancy.

The element contents of the residues were measured by energy dispersive X-ray spectroscopy. As shown in Table 6, the residue of neat PA6 was composed of a high content of carbon and relatively low content of oxygen. For the residue of PA6/AlPi-13, the carbon content decreased dramatically, and aluminum and phosphorus were observed on the char layer, which further reveals that AlPi also acts in condensed phase [55]. With the incorporation of 1.5 wt.% DES, the contents of oxygen, aluminum, and phosphorus on the char layer increased simultaneously, suggesting that the aromatic Schiff base groups of DES promote the formation of aluminum phosphorus oxides and the derivatives in condensed phase [59], leading to heavier char residue and improved flame retardancy. 

**Figure 6:**
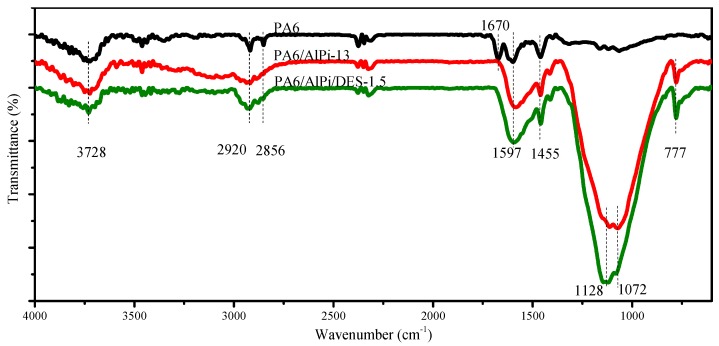
FTIR spectra of the residues after the cone calorimeter test.

Furthermore, FTIR spectrometry was used to investigate the chemical composition of the char residues. As shown in Figure 6, the curve of neat PA6 shows the characteristic absorption peaks at 3728 cm^−1^ (N–H); 2920, 2856 and 1455 cm^−1^ (C–H stretching and bending vibrations) [59]; 1670 cm^−1^ (C=O) [33]; and 1597 cm^−1^ (C=C stretching bands of aromatic ring) [44]. For PA6/AlPi-13, the main characteristic peaks mentioned above were almost reproduced. The peak at 1597 cm^−1^ increased obviously, indicating that AlPi promotes the aromatization of PA6 molecular chains in the char layer [55]. Conspicuous additional peaks appeared from 1072 to 1128 cm^−1^, which can be attributed to aluminum phosphorus oxides, aluminum polyphosphates, or pyrophosphates [60]. The signals of phosphorus- and aluminum-based compounds were too strong and overlapped with each other. The peak at 777 cm^−1^, which is assigned to the phosphorus oxides and their derivatives, was observed [59]. Compared to PA6/AlPi-13, the residue of PA6/AlPi/DES-1.5 exhibited similar characteristic peaks. Additionally, the absorption peaks from 1072 to 1128 cm^−1^ enhanced significantly, revealing that the contents of aluminum phosphorus oxides and polyphosphates increased. Combined with the analysis of SEM, element contents, and the FTIR spectrometer, it should be concluded that DES fixes the phosphorous and aluminum species in the residues and promotes the formation of the char layer effectively in the condensed phase.

### 3.6. Thermal Degradation

**Figure 7:**
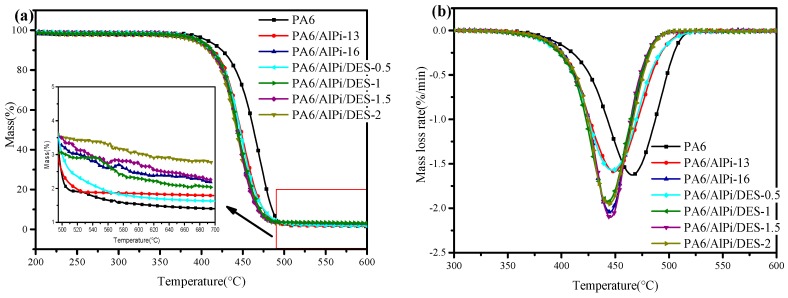
(**a**) TGA and (**b**) DTG curves of neat PA6 and flame-retarded PA6.

To investigate the thermal degradation process of PA6/AlPi/DES composites, thermogravimetric analysis was performed under a nitrogen atmosphere. The corresponding TGA and differential thermogravimetry (DTG) results are presented in Figure 7a,b. *T_5%_*, the temperature at the maximum decomposition rate (*T_max_*), and the residue weight at 700 °C are summarized in Table S2. 

As shown in Figure 7, all the specimens presented one step during the thermal degradation process. With the addition of AlPi, the *T_5%_* of PA6/AlPi-13 decreased from 406 °C of neat PA6 to 392 °C, and the *T_max_* of PA6/AlPi-13 decreased from 466 °C of neat PA6 to 446 °C, which is attributed to AlPi catalyzing the degradation of PA6 and then reducing the thermal stability of the PA6/AlPi composites [26]. Additionally, the *T_5%_* and *T_max_* of all the specimens remained almost unchanged with the increasing DES contents, suggesting that DES did not reduce the thermal stability of PA6 composites. With the increasing amount of DES, the residues at 700 °C increases from 1.6% of PA6/AlPi/DES-0.5 to 2.8% of PA6/AlPi/DES-2, which is attributed to the formed char layer promoted by DES.

**Figure 8:**
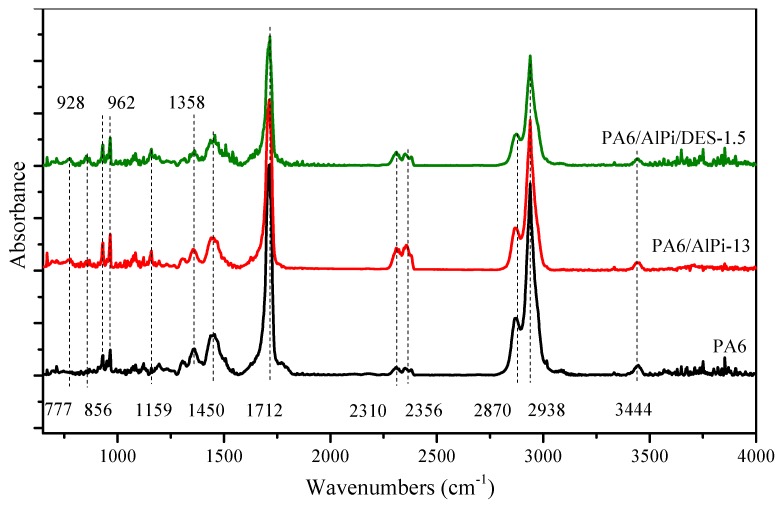
FTIR spectra of pyrolysis gases of PA6 and flame-retarded PA6 at maximum weight loss rate.

To further analyze the flame-retarded mechanism of the composites during the thermal decomposition process, TGA-FTIR analysis was performed, and the FTIR spectra of the pyrolysis gases at *T_max_* are presented in Figure 8. The characteristic bands of the major degradation products of PA6 can be identified in all spectra, such as aliphatic hydrocarbons (2870, 2938 and 1450 cm^−1^), ε-caprolactam (1712 and 1358 cm^−1^), CO_2_ (2356 and 2310 cm^−1^), and NH_3_ (3442, 962 and 928 cm^−1^) [23,59,61,62]. The spectra of PA6/AlPi composites exhibited additional characteristic bands of P=O (1159 cm^−1^), P–O–P (856 cm^−1^), and PO^−^ anion (777 cm^−1^) [25], as a result of the gaseous-phase flame-retarding mechanism of AlPi. For the spectrum of PA6/AlPi/DES-1.5, no additional peak was identified compared with PA6/AlPi composites, indicating that the role of DES in gas phase was not evident.

**Figure 9:**
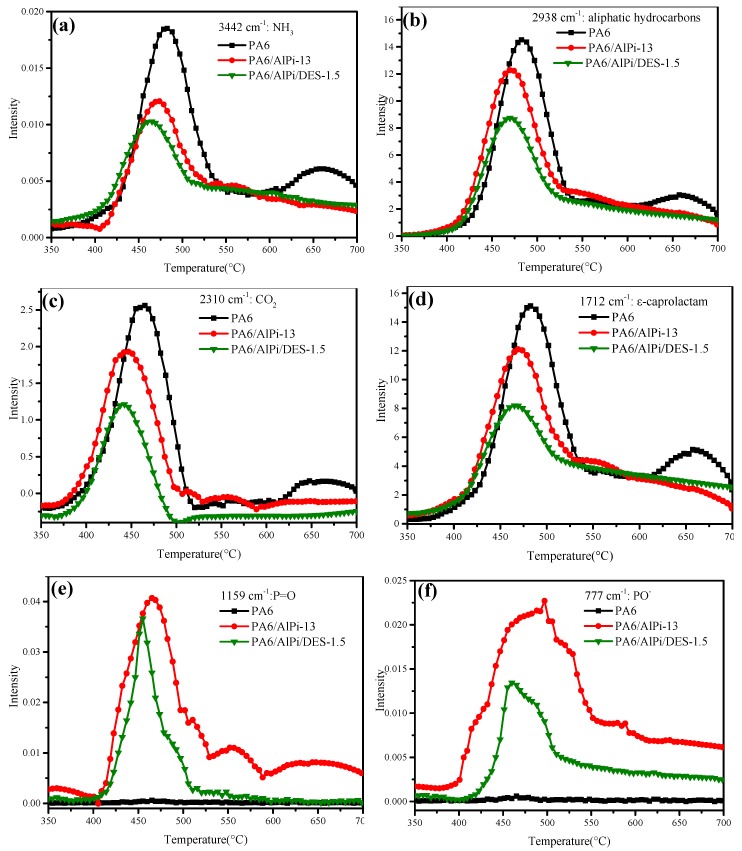
Release rates of (**a**) NH_3_, (**b**) aliphatic hydrocarbons, (**c**) CO_2_, (**d**) ε-caprolactam, (**e**) P=O, and (**f**) PO^−^ emitted by degradation of PA6 and flame-retarded PA6.

To figure out the influences of DES and AlPi on the flame-retarded mechanism of the composites, the release rates of pyrolysis gases at different temperatures were estimated by quantifying the intensities of their corresponding absorption peaks. The characteristic peaks at 3442 cm^−1^ (NH_3,_ Figure 9a), 2938 cm^−1^ (C–H of aliphatic hydrocarbons, Figure 9b), 2310 cm^−1^ (CO_2_, Figure 9c), 1712 cm^−1^ (C=O of ε-caprolactam, Figure 9d), 1159 cm^−1^ (P=O, Figure 9e) and 777 cm^−1^ (PO^−^, Figure 9f) were investigated. With the addition of AlPi, all the release rates of major pyrolysis products from PA6 (NH_3_, aliphatic hydrocarbons, CO_2_, and ε-caprolactam) were clearly reduced. PA6/AlPi composites presented lower temperatures at the maximum release rate, which is consistent with the result from TGA analysis. Furthermore, the addition of DES reduced the intensities of the major degradation products mentioned above, indicating that more substances remained in the condensed phase to form the char barrier. The decreasing combustible volatiles like aliphatic hydrocarbons and ε-caprolactam also contributed to the flame retardancy. 

Additionally, as shown in Figure 9e,f, phosphorus-containing pyrolysis products (P=O and PO^−^) were detected from the pyrolysis gases of PA6/AlPi-13. During the thermal decomposition process, AlPi was further degraded and released phosphoric free radicals, which acted as scavengers and quenched the radicals in the gaseous phases and intercepted the chain reactions during combustion [63]. The presence of DES reduced the release rates and the total release amounts of P=O and PO^−^, just like the other pyrolysis products, demonstrating a synergistic effect with AlPi in the condensed phase.

## 4. Conclusions

The diepoxide DES demonstrated efficient chain extension effect on PA6 through the coupling reaction of the epoxide groups and the end-groups of PA6 molecular chains. The addition of 2 wt.% DES improved the equilibrium melt torque by more than 50% based on neat PA6. A small amount of DES prevented the mechanical deterioration induced by AlPi, and enhanced the mechanical properties of AlPi/PA6 composites significantly. Compared with those of the V-0 flame-retarded PA6 without DES, the tensile, flexural, and Izod notched impact strengths presented enhancements of 16.2%, 16.5%, and 24.9%, respectively. The aromatic Schiff base groups in DES brought about significant improvement in the flame retardancy of AlPi/PA6 composites. PA6/AlPi/DES-1.5 achieved a UL-94 V-0 rating at 0.8 mm thickness, with a high LOI value of 34.9%, indicating that the synergistic effect of AlPi and DES afforded efficient flame retardancy properties. Compared to neat PA6, 1.5 wt.% DES reduced the peak HRR by 37%, but increases the residual mass by 85% in the cone calorimetric test. The elemental and infrared characterization of the char residues revealed that DES promotes the formation of aluminum phosphorus oxides and polyphosphates in the residues and acts in the condensed-phase flame retardation. These results prove that aromatic Schiff base derivatives have great potential in flame retardant applications.

## Figures and Tables

**Table 1:** The composition of neat polyamide 6 (PA6) and PA6 composites.

Samples	Composition (wt.%)		
	PA6	AlPi	DES
PA6	100	—	—
PA6/DES-1	99	—	1
PA6/DES-2	98	—	2
PA6/DES-3	97	—	3
PA6/DES-4	96	—	4
PA6/DES-5	95	—	5
PA6/AlPi-13	87	13	—
PA6/AlPi-16	84	16	—
PA6/AlPi/DES-0.5	86.5	13	0.5
PA6/AlPi/DES-1	86	13	1
PA6/AlPi/DES-1.5	85.5	13	1.5
PA6/AlPi/DES-2	85	13	2

**Table 2:** Mechanical properties and melt flow rates of neat PA6 and PA6 composites. ^1^

Samples	Mechanical Properties			Melt Flow Rate [g/(10 min)]
	Izod Notched Impact Strength (kJ/m^2^)	Tensile Strength (MPa)	Flexural Strength (MPa)	
PA6	6.6 ± 0.2	59.5 ± 1.0	89.8 ± 1.2	17.2 ± 0.4
PA6/AlPi-13	6.4 ± 0.3	56.4 ± 0.9	86.1 ± 1.0	11.8 ± 0.3
PA6/AlPi-16	5.8 ± 0.1	55.0 ± 0.9	83.8 ± 0.8	12.1 ± 0.4
PA6/AlPi/DES-0.5	6.9 ± 0.2	60.6 ± 1.2	90.6 ± 0.7	7.2 ± 0.2
PA6/AlPi/DES-1	7.2 ± 0.1	63.3 ± 1.0	93.3 ± 0.9	3.5 ± 0.2
PA6/AlPi/DES-1.5	7.2 ± 0.3	63.9 ± 0.9	97.6 ± 1.0	2.3 ± 0.2
PA6/AlPi/DES-2	7.3 ± 0.2	61.9 ± 1.2	95.6 ± 1.0	2.3 ± 0.1

^1^ The results are averaged from 5 specimens.

**Table 3:** Melting temperature (*T_m_*), melting enthalpy (Δ*H_m_*), and crystallinity (*X_c_*) of neat PA6 and flame-retarded PA6.

Samples	*T_m_* (°C)	Δ*H_m_* (J/g)	*W_f_* (%)	*X_c_* (%)
PA6	223.5	49.9	100	26.2
PA6/AlPi-13	222.5	43.5	87	26.3
PA6/AlPi-16	223.0	37.8	84	23.7
PA6/AlPi/DES-0.5	223.0	42.3	86.5	25.8
PA6/AlPi/DES-1	223.0	40.2	86	24.6
PA6/AlPi/DES-1.5	223.5	39.1	85.5	24.0
PA6/AlPi/DES-2	221.9	37.3	85	23.1

**Table 4:** LOI values and UL-94 test results of neat PA6 and flame-retarded PA6 composites. ^1^

Samples	LOI (%)	UL-94				
		Thickness (mm)	*T_1_*^2^ (s)	*T_2_*^2^ (s)	Burning Drips	Rating
PA6	24.2	1.6	>60	>60	Yes	NR
PA6/DES-2	25.5	1.6	>60	>60	Yes	NR
PA6/AlPi-13	29.3	0.8	42	>60	Yes	NR
		1.6	2	2	Yes	V-2
PA6/AlPi-16	31.8	0.8	2	5	Yes	V-2
		1.6	1	1	No	V-0
PA6/AlPi/DES-0.5	32.2	0.8	10	3	Yes	V-2
		1.6	2	2	No	V-0
PA6/AlPi/DES-1	33.5	0.8	3	4	Yes	V-2
		1.6	1	2	No	V-0
PA6/AlPi/DES-1.5	34.9	0.8	2	2	No	V-0
		1.6	1	1	No	V-0
PA6/AlPi/DES-2	35.4	0.8	3	1	No	V-0
		1.6	2	1	No	V-0

^1^ The results are averaged from 5 specimens. NR: no rating; ^2^ Average combustion duration after the first (*T_1_*) and the second ignition (*T_2_*).

**Table 5:** Cone calorimeter parameters of neat PA6 and flame-retarded PA6.

Samples	TTI (s)	Peak HRR (kW/m^2^)	THR (MJ/m^2^)	Peak RSR ((m^2^/s)/m^2^)	TSR (m^2^/m^2^)	Residue (wt.%)
PA6	248	759.2	121.8	1.5	144.3	3.6
PA6/AlPi-13	230	536.5	123.9	9.8	1677.1	5.1
PA6/AlPi/DES-1.5	228	478.0	126.6	7.5	1620.3	6.7

**Table 6:** Element contents of the residues after the cone calorimeter test. ^1^

Samples	Element Content (wt.%)			
	C	O	Al	P
PA6	81.2 ± 0.8	18.8 ± 0.8	—	—
PA6/AlPi-13	37.7 ± 1.2	40.8 ± 3.1	8.6 ± 0.7	12.9 ± 1.5
PA6/AlPi/DES-1.5	27.2 ± 1.6	47.2 ± 1.8	9.5 ± 0.6	16.0 ± 1.3

^1^ The average and standard deviation values were obtained from 5 measurements. Nitrogen content could not be detected accurately with the instrument used.

## Supplementary Materials

The following are available online at https://www.mdpi.com/1996-1944/12/14/2217/s1, Figure S1: ^1^H NMR spectrum of bis(4-formylphenyl) ether in CDCl_3_; Figure S2: ^1^H NMR spectrum of compound 5 in CDCl_3_; Figure S3: ^1^H NMR spectrum of DES in DMSO-d_6_; Figure S4: FTIR spectrum of DES; Figure S5: TGA and DSC curves of DES; Table S1: Carboxyl and amino end-group concentration of chain-extended PA6 with different DES contents; Table S2: The initial decomposition temperature at 5% weight loss (*T_5%_*), the temperature at the maximum decomposition rate (*T_max_*), and the residue weight at 700 °C of neat PA6 and flame-retarded PA6.
