# Supplementary material for: Chain Extension and Synergistic Flame-Retardant Effect of Aromatic Schiff Base Diepoxide on Polyamide 6/Aluminum Diethylphosphinate Composites
Source: Materials (Basel). 2019 Jul 10;12(14):2217. doi: 10.3390/ma12142217 (PMC6678687; doi:10.3390/ma12142217)
Supplement: Supplementary file 1 [file materials-12-02217-s001.pdf]

Supplementary Materials: Chain Extension and Synergistic Flame-Retardant Effect of Aromatic Schiff Base Diepoxide on Polyamide 6/Aluminum Diethylphosphinate Composites

Tianxiang Liang [1,2], Jianan Cai [1], Shumei Liu [1], Hualin Lai [2,*] and Jianqing Zhao [1,*]

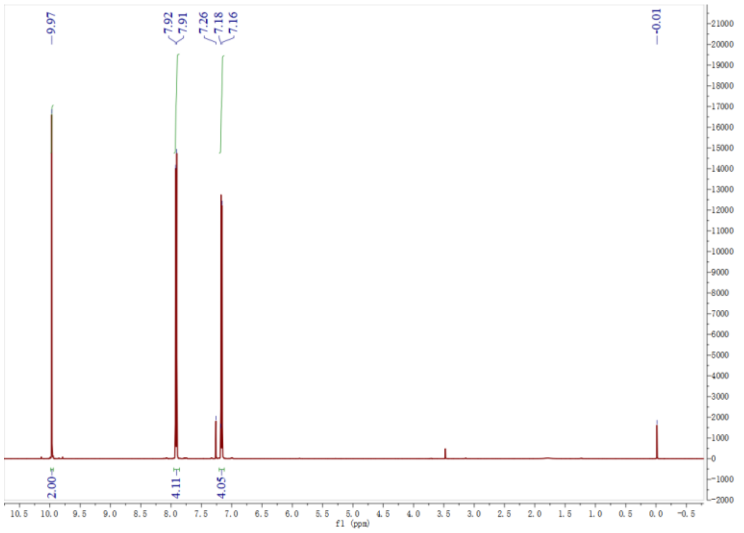

Figure S1. ^{1}H NMR spectrum of bis(4-formylphenyl) ether in $CDCl_3$.

^{1}H NMR (600 MHz, Chloroform-*d*, δ, ppm): 9.97 (s, 2 H, –CHO), 7.92 (d, 2H, Ar–H), 7.81 (d, 2H, Ar–H).

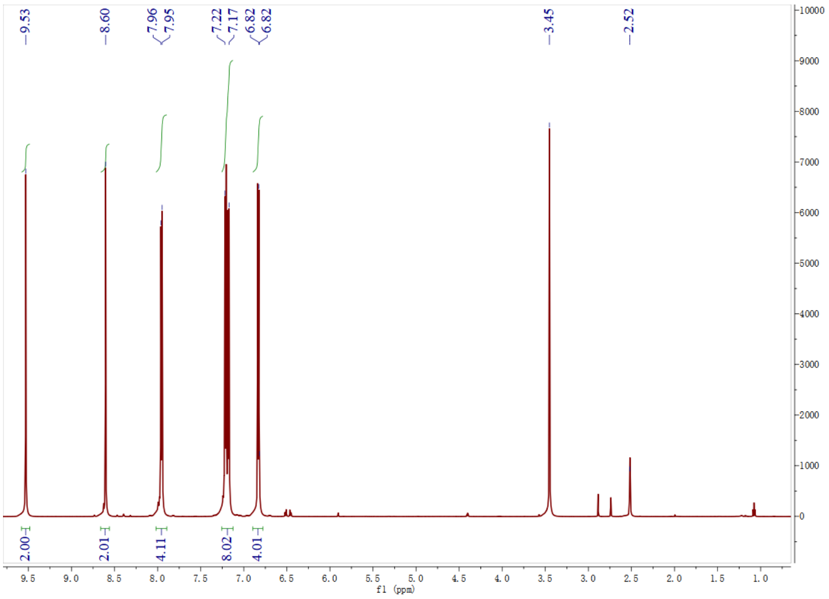

Figure S2. ^{1}H NMR spectrum of compound 5 in $CDCl_3$.

^{1}H NMR (600 MHz, DMSO-*d6*, δ, ppm): 9.53 (s, 2 H, –OH), 8.60 (s, 2H, –C=N–), 7.95 (d, 4H, Ar–H), 7.22 (d, 4H, Ar–H), 7.17 (d, 4H, Ar–H), 6.82 (d, 4H, Ar–H).

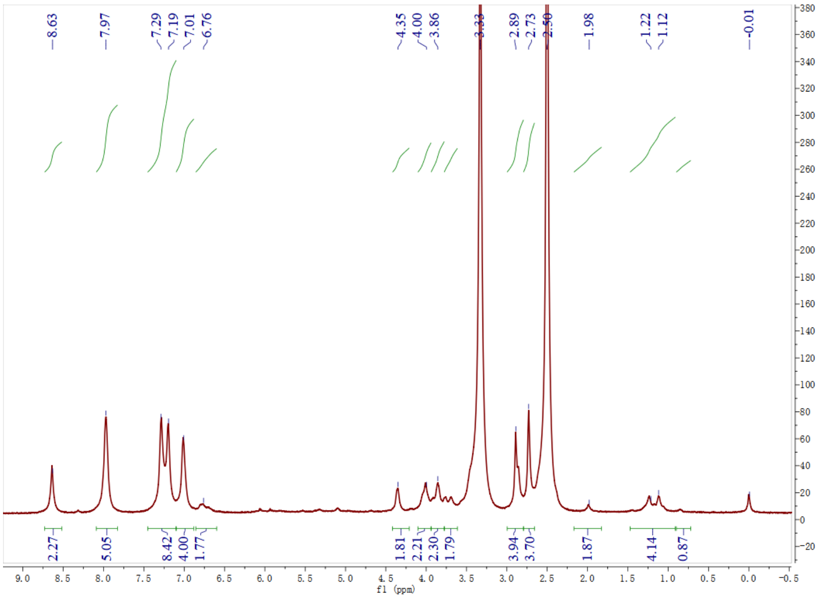

Figure S3. ^{1}H NMR spectrum of DES in DMSO-d_6.

^{1}H NMR (600 MHz, Chloroform-*d*, δ, ppm): 8.63 (s, 2 H, –C=N–), 7.97 (d, 4H, Ar–H), 7.29 (d, 4H, Ar–H), 7.19 (d, 4H, Ar–H), 6.76 (d, 4H, Ar–H), 4.35 (d, 2H, CH), 4.20–3.75 (m, 4H, –CH_2-), 2.90–2.70 (m, 4H, –CH_2–).

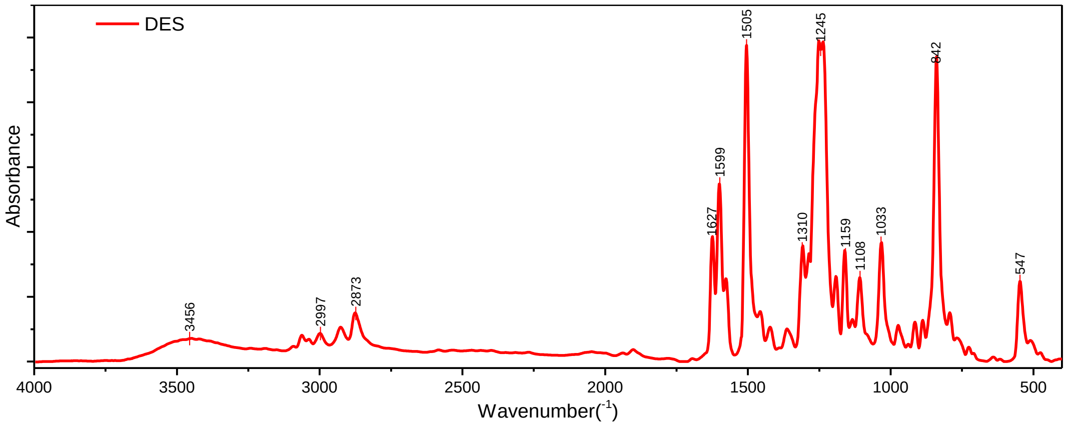

Figure S4. FTIR spectrum of DES.

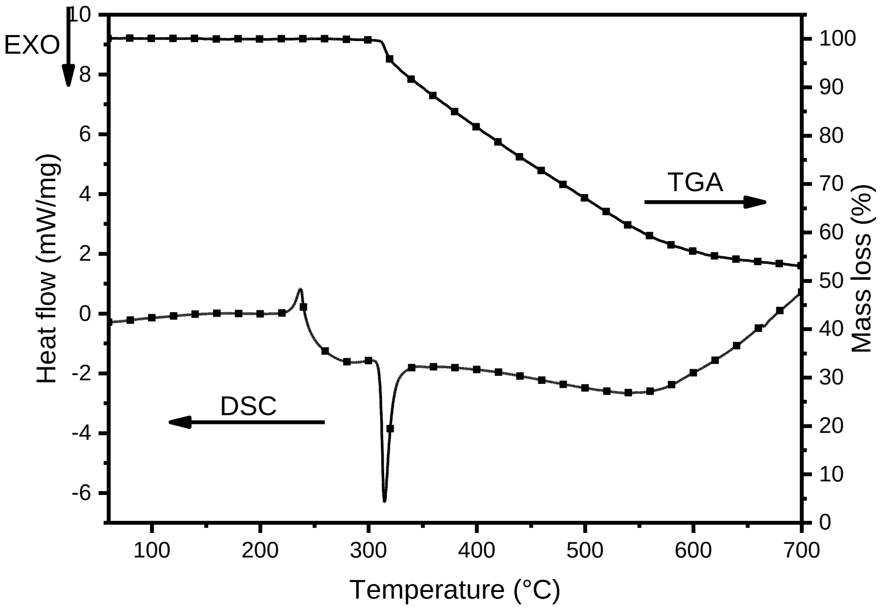

Figure S5. TGA and DSC curves of DES.

Table S1. Carboxyl and amino end-group concentration of chain-extended PA6 with different DES contents.

DES Content (wt. %)	Carboxyl End-Group Concentration (μmol/g)	Amino End-Group Concentration (μmol/g)
**0**	239.84	37.23
**1**	166.95	34.43
**3**	128.30	29.63
**5**	80.45	24.21

Table S2. The initial decomposition temperature at 5% weight loss ($T_{5\%}$), the temperature at the maximum decomposition rate (T_{max}), and the residue weight at 700 °C of neat PA6 and flame-retarded PA6.

Samples	$T_{5\%}$ (°C)	T_{max} (°C)	Char Residue (wt. %) (700 °C)
PA6	406	466	1.4
PA6/AlPi-13	392	446	1.8
PA6/AlPi-16	394	444	2.2
PA6/AlPi/DES-0.5	395	442	1.6
PA6/AlPi/DES-1	393	445	2.1
PA6/AlPi/DES-1.5	395	444	2.3
PA6/AlPi/DES-2	396	444	2.8
